# Structure Characterization and Immunomodulatory Activity of *Misgurnus anguillicaudatus* Carbohydrates

**DOI:** 10.3390/molecules28155771

**Published:** 2023-07-31

**Authors:** Liyuan Yun, Conglin Han, Xiaoqing He, Qian Li, Viktor Fersht, Min Zhang

**Affiliations:** 1 Key Laboratory of Smart Breeding (Co-Construction by Ministry and Province), Ministry of Agriculture and Rural Affairs, Tianjin 300392, China; yunly1120@163.com (L.Y.); limengqia@163.com (Q.L.); 2 China-Russia Agricultural Processing Joint Laboratory, Tianjin Agricultural University, Tianjin 300392, China; 18722212017@163.com (C.H.); hhemily@163.com (X.H.);; 3 State Key Laboratory of Food Nutrition and Safety, Tianjin University of Science & Technology, Tianjin 300457, China

**Keywords:** *Misgurnus anguillicaudatus*, carbohydrates, structure, immune activity

## Abstract

*Misgurnus anguillicaudatus,* also known as oriental weather loach, is widely consumed and favored in East Asia due to its superior nutritional values and excellent flavor. In this study, a crude *Misgurnus anguillicaudatus* carbohydrates (MAC) was isolated from *Misgurnus anguillicaudatus*. Subsequently, two parts, which were named MAO and MAP, respectively, were separated from MAC, and their primary structures and immunomodulatory activity were investigated. The results showed that MAO had a molecular weight of 2854 Da, and principally consisted of arabinose (77.11%) and rhamnose (21.97%), together with minor levels of fucose (0.92%); MAP, with a molecular weight of 3873 Da, was mainly composed of fucose (87.55%) and a small amount of rhamnose (8.86%) and galactose (3.59%). The in vitro assay showed that MAC could significantly enhance the proliferation of macrophages without cytotoxicity and increase the production of immune substances (TNF-α, IL-6). Together with Western blot results, we speculated that MAC could stimulate RAW264.7 murine macrophage cells to secrete TNF-α and IL-6 through up-regulating TLR4-MAPK-p38 signaling pathways. The results indicated that MAC could be a potential immune agent and might provide meaningful information for further chain conformation and immune mechanism research.

## 1. Introduction

Polysaccharides are macromolecules that commonly exist in plants, animals and microorganisms and generally have a wide range of bioactivities with low toxic and side effects [1]. Moreover, polysaccharides are also known to affect a variety of biological responses, especially the immune response [2]. Some polysaccharides could be recognized and combined with specific receptors on macrophages to enhance the viability of macrophages against pathogenic microorganisms and tumorigenesis by the promotion of phagocytic and the cytokines of IL-6 and TNF-α secretion. Then, it has been thought that the polysaccharides with immunomodulatory activity could be good immunomodulator [3,4]. It is worth noting that macrophages play a unique role in the immune system. Not only do they initiate innate immune responses, they also help fight infections and inflammation. Macrophages can kill pathogens directly by phagocytosis and indirectly by secreting cytokines such as TNF-α and IL-6 [5,6]. Therefore, macrophages are an ideal cell model for evaluating the immunomodulatory activity of bioactive substances.

Loach (*Misgurnus anguillicaudatus*), which contains protein, fat, carbohydrate, vitamins and some inorganic elements, is a common freshwater fish in China, Japan, and Korea due to its high nutritional and market value [7]. It has been known to Chinese since ancient times because of its good taste and flavor. Therefore, *Misgurnus anguillicaudatus* has become an important freshwater aquaculture species for several decades in China [8]. In recent years, numerous studies focus on reporting the activity of *Misgurnus anguillicaudatus* peptide, such as antioxidant activity, antiproliferative activities, and anti-fatigue effect [9]. In the report of Zhang et al., they separated four parts polysaccharides from *Misgurnus anguillicaudatus* and investigated the antioxidation and antiglycation of polysaccharides [10]. Qin Chuanguang et al. isolated two parts polysaccharides from *Misgurnus anguillicaudatus,* and the average molecular weight was 1.30 × 10^5^ and 1.23 × 10^5^, respectively [11]. However, little information of polysaccharides from *Misgurnus anguillicaudatus* is known especially regarding their methods of purification, their chemical characteristics and immunologicalactivities.

Therefore, in this present study, a crude *Misgurnus anguillicaudatus* carbohydrates (MAC) was isolated from *Misgurnus anguillicaudatus* using hot water extraction, and two parts named as *Misgurnus anguillicaudatus* oligosaccharides (MAO) and *Misgurnus anguillicaudatus* polysaccharides (MAP) were purified from MAC by the DEAE-52 cellulose chromatography. The primary chemical structure of MAO and MAP was characterized by high-performance liquid chromatography (HPLC), and gas chromatography-mass spectrometer (GC-MS). The potential effect of MAC on immune regulatory activities in murine Raw 264.7 macrophages was also investigated. The results of this study may provide useful information for further study of *Misgurnus anguillicaudatus.*

## 2. Results and Discussion

### 2.1. Homogeneity and Molecular Weight Analysis

**Figure 1:**
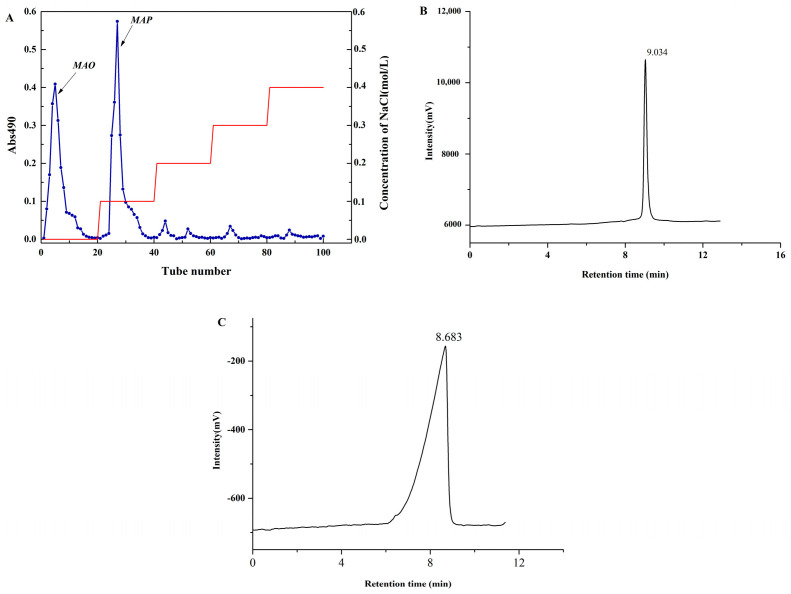
Chromatogram of the polysaccharides from *Misgurnus Anguillicaudatus* by DEAE chromatography (**A**), HPLC of MAO (**B**), and HPLC of MAP (**C**).

As shown in Figure 1A, there were two major components eluting with distilled water and 0.1 mol/L NaCl solution, respectively. The two elution fractions were collected, dialyzed and lyophilized to obtain purified polysaccharide. There were two sole and sharp symmetrical peaks on the HPLC chromatogram (Figure 1B,C).

The polydispersity index (Mw/Mn) was 1.35 and 1.41, respectively, which also demonstrated that the two parts were relatively homogeneous polysaccharides after separation and purification was carried out by DEAE-52 cellulose chromatography. The molecular weight is shown in Table 1. According to the molecular weight, it can be speculated that the two parts are *Misgurnus anguillicaudatus* oligosaccharides (MAO, in Figure 1B) and *Misgurnus anguillicaudatus* polysaccharides (MAP, in Figure 1C), respectively. MAO was the eluted component with distilled water, and MAP was the eluted component with 0.1 mol/L NaCl solution. The total carbohydrate contents of MAO and MAP were 94.43% and 98.80%. However, the results of Zhang et al. [10] found four polysaccharides (the viscoaverage molecular weight was 181.94 kDa, 169.32 kDa, 117.51 kDa, and 221.41 kDa) from *Misgurnus anguillicaudatus* (MAPs). In the research of Qin Chuanguang et al., they also found two major constituents from *Misgurnus anguillicaudatus* by using Sephadex G-100 column gel filtration [11]. That may contribute to the difference between the extraction and separation methods of polysaccharides.

The content of protein, total sugars and glucuronic acid in MAC, MAO, and MAP is shown in Table 2. The content of protein in MAO and MAP is not detected by the Coomassie bright blue method.

### 2.2. Monosaccharide Composition Analysis

**Figure 2:**
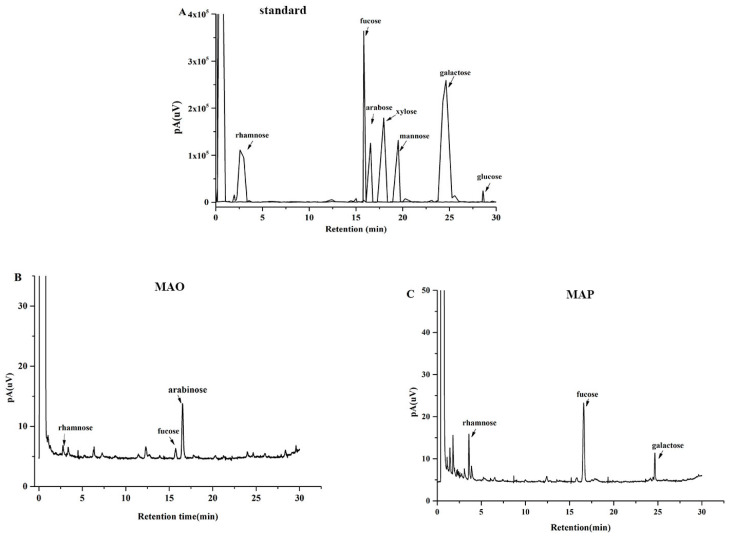
Gas chromatography analysis of monosaccharide standard mixture (**A**); the monosaccharide composition of MAO (**B**) and MAP (**C**).

The chromatograms for monosaccharide composition of the two fractions are shown in Figure 2. MAO was principally comprised with arabinose (77.11%), rhamnose (21.97%), and fucose (0.92%). In addition, the arabinose ratio was more than 70%, suggesting that the backbone of MAO may be composed of arabinose. MAP was mainly composed of fucose (87.55%), rhamnose (8.86%), and galactose (3.59%). Qin chuanguang et al. reported that *Misgurnus anguillicaudatus* polysaccharides contain 40% fucose and its average molecular weight was 1.30 × 10^5^ in their study [11]. This phenomenon may be due to the different extraction and separation methods, raw material origin varieties, etc.

### 2.3. Fourier Transform Infrared (FT-IR) Spectrum

**Figure 3:**
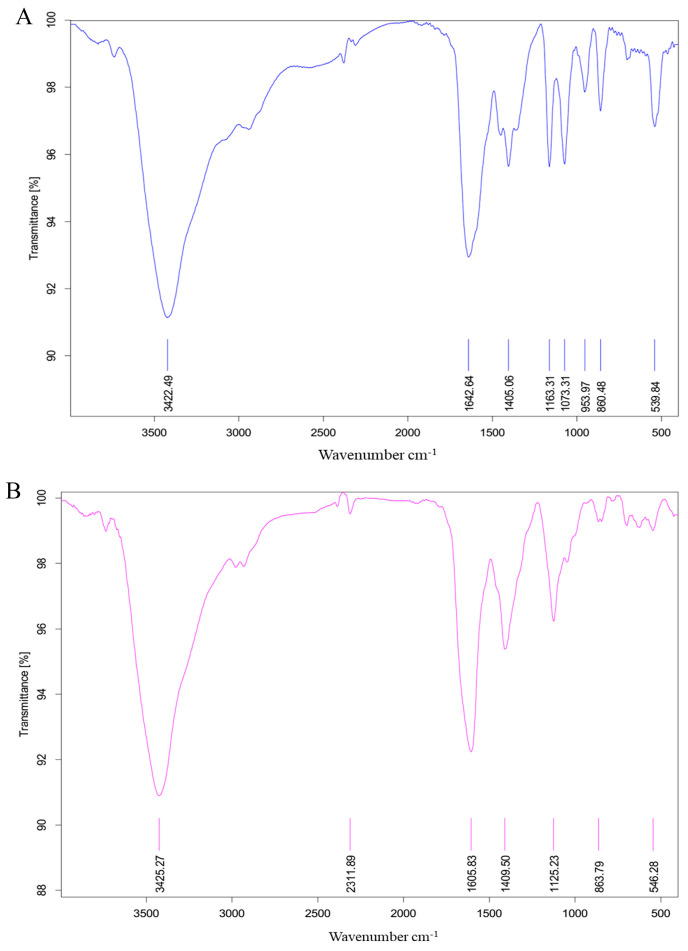
FT-IR spectrum of MAO (**A**) and MAP (**B**).

The FT-IR spectrum of MAO is shown in Figure 3A. The absorbance bands at 3422.49 cm^−1^ belonged to O-H stretching vibration. The absorbance bands at 1642.64 cm^−1^ and 1405.06 cm^−1^ were due to the asymmetric absorption vibration of C=O bond [12]. The absorbance bands at 1163.31 cm^−1^ and 1073.3 cm^−1^ are caused by C-O vibration, indicating that there is C-O-C. Absorption peaks between 1000 cm^−1^ and 1200 cm^−1^ suggest the pyranose ring of sugar residues [13]. The absorbance bands at 860.48 cm^−1^ proposed a α-glycosidic bond was simultaneous [14].

The FT-IR spectrum of MAP is shown in Figure 3B. The broad peak at 3425.27 cm^−1^ is the characteristic peak of hydrogen bounded O-H stretching vibration. The absorbance bands at 1605.83 cm^−1^ and 1409.50 cm^−1^ may be caused by C=O stretching vibration [15], and C=C stretching vibration, respectively. The prominent band at 1125.23 cm^−1^ is characterized by overlapping of C-O-C glycoside bond vibration and ring vibration with the telescopic vibration of the side-group C-O-H bond boundary [16]. Moreover, the characteristic absorption at 863.79 cm^−1^ in the FT-IR spectra indicated that α-configuration was simultaneous [17]. The α-configuration of MAP was in accordance with the result of Qin Chuanguang et al. [11].

### 2.4. NMR Spectra (^13^C NMR) for MAO and MAP

**Figure 4:**
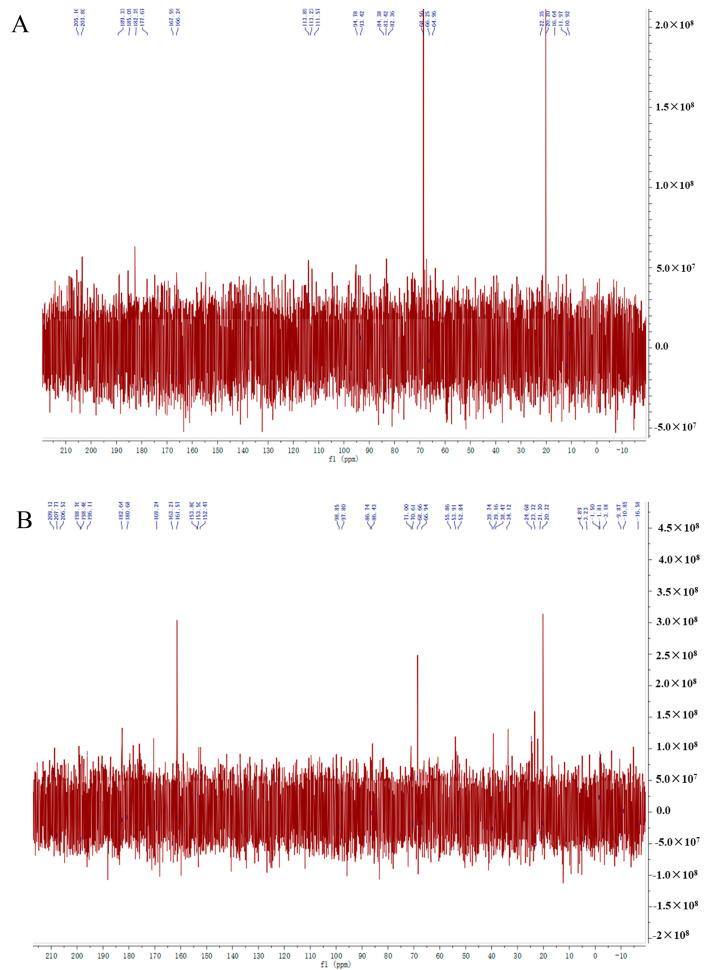
^13^C NMR spectrum of MAO (**A**)and MAP (**B**).

The MAOS spectra (Figure 4A) showed no signal peak at δ160–180 ppm, indicating no uronic acid, which was a neutral sugar. MAP spectra (Figure 4B) peak at δ160–180 ppm, indicating the presence of uronic acid, which is an acidic sugar. According to Sulphate-carbazole method, the content of uronic acid in MAO was not detected, and in MAP was 1.07% ± 0.02%. Above these results, we speculated that MAO was a neutral sugar, and MAP was an acidic sugar.

### 2.5. Scanning Electron Microscope (SEM) Morphology Observation

**Figure 5:**
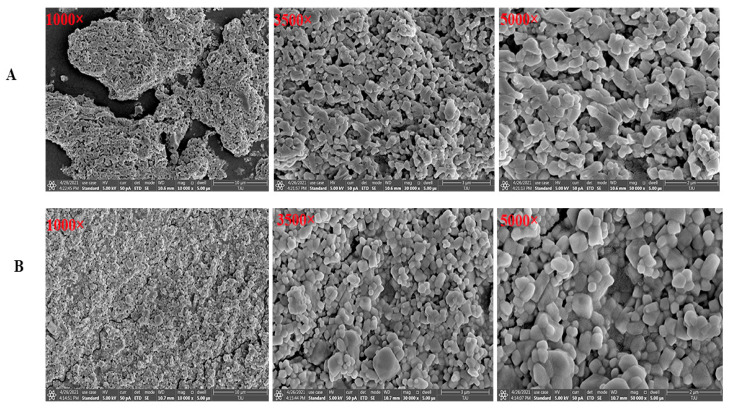
Scanning electron microscope (SEM) morphology observation of MAO (**A**) and MAP (**B**).

The digital photographs of MAO and MAP are illustrated in Figure 4B and Figure 5A. The texture of MAO with irregular structures such as interaction and small pieces of fragments were shown on the surface. In comparison to that of MAO, the SEM images of MAP (Figure 5B) indicated that MAP entangled to form several irregular shapes that are tightly bound.

### 2.6. Conformational Structure of MAO and MAP

**Figure 6:**
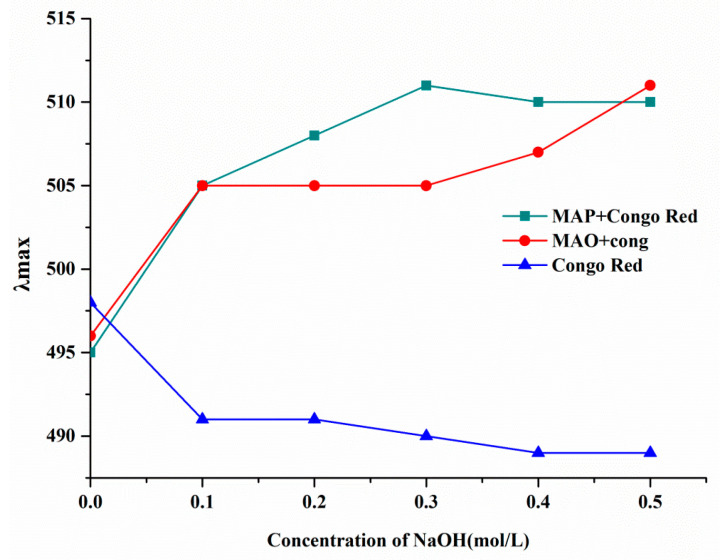
Triple helical conformation analysis of MAO and MAP.

Congo red reacts with triple-helix polysaccharides to shift the maximum absorption peak towards longer wavelengths in solution. The transition from a triple-helix conformation to a single coil conformation decreases the maximum absorption of the Congo Red-polysaccharides solution [18]. According to previous reports, polysaccharides with low molecular weights might have a triple helical structure [19,20]. In the research of Yun Wang et al., they found that ginger polysaccharide 1 (GP1), which had a molecular weight of 6128 Da, had a three-helix structure [21]. Therefore, the triple helical structures of MAO and MAP were further analyzed in this study. As shown in Figure 6, compared with Congo red, a bathochromic shift (from 495 nm to 510 nm) was shown in the MAO–Congo red complex and the MAP–Congo red complex with the NaOH concentration increasing, but there was no significant decline, which indicated that neither MAO or MAP has a helical structure. This result was similar to that reported by Qin Chuanguang et al. [11].

### 2.7. Effects of MAC on RAW264.7 Cell Viability

Polysaccharides, with immune activity, could exhibit various beneficial pharmacological effects via the ability to modulate macrophage immune function [22].

**Figure 7:**
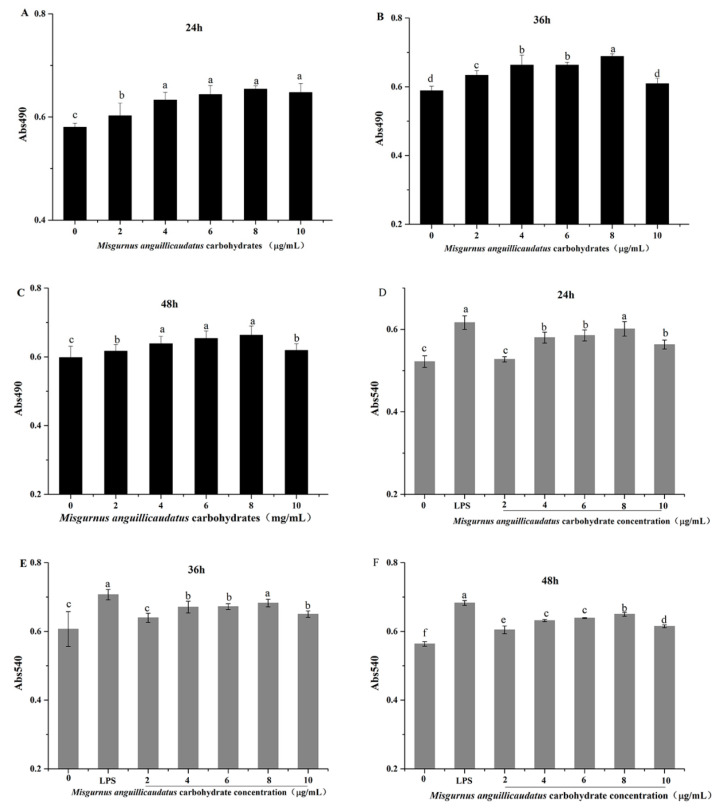
Effect of MAC on the viability of RAW 264.7 cells for different periods of time (24 h (**A**), 36 h (**B**), and 48 h (**C**)). Effect of MAC on taking neutral red of RAW 264.7 cell for 24 h (**D**), 36 h (**E**), and 48 h (**F**). The value was expressed as the mean ± SD, n = 6. The different letters indicate significant differences, *p* < 0.05.

As shown in Figure 7, MAC showed a promotive effect on proliferation of RAW264.7 cells in the range of 2 μg/mL to 10 μg/mL for 24 h to 48 h, and significant differences were observed compared with blank controls (*p* < 0.05). The viability of RAW264.7 cells treated with MAC (2–10 μg/mL) for 36 h was significantly higher than other two treatments.

### 2.8. Effects of MAC on RAW264.7 Cells Phagocytic Activity

Phagocytosis invading pathogens plays a significant role in activating the immune function of macrophages, and the immune function of macrophages can be evaluated by their phagocytic activity [23]. Therefore, we used neutral red test to evaluate whether MAC could promote the phagocytosis of RAW264.7 cells. As shown in Figure 7D–F, the phagocytic ability of RAW264.7 cells treated with MAC (2 μg/mL–10 μg/mL) was higher than that of the control group (0 μg/mL). In addition, the phagocytic activity of 8 μg/mL MAC-treated cells was similar to that of lipopolysaccharide (LPS) group (*p* > 0.05), while the phagocytosis activity showed a downward trend when the MAC concentration increased to 10 μg/mL At the same time, the phagocytosis activity of cells treated with MAC (2 μg/mL–10 μg/mL) for 36 h was higher than 24 h and 48 h. Therefore, according to the above experiment results, the MAC (2 μg/mL–10 μg/mL) treated cell for 36 h were selected for further experiments. The function of phagocytes were enhanced by MAC which increasing the body’s resistance to foreign substances such as pathogens and tumor cells [24]. These results indicated that MAC can induce macrophages to enhance phagocytosis.

Notably, molecular weight had discrepant immune regulation effects in different substances. The molecular weight of polysaccharides had discrepant immune regulation effects in different substances [25]. Lower-molecular-weight polysaccharides have a simpler structural conformation, conferring a certain dominance that passes through the cell barrier with less hinderance. Ting Zhao et al. reported that Schisandra polysaccharide (SCPP11), which has average molecular weight of 3.4 × 10^3^ Da, exerted its antitumor activity by improving immune system functions [26]. Therefore, in this study, the molecular weight may be one of the important factors to the MAC immune activity.

### 2.9. Effects of MAC on RAW264.7 Cells Morphology

**Figure 8:**
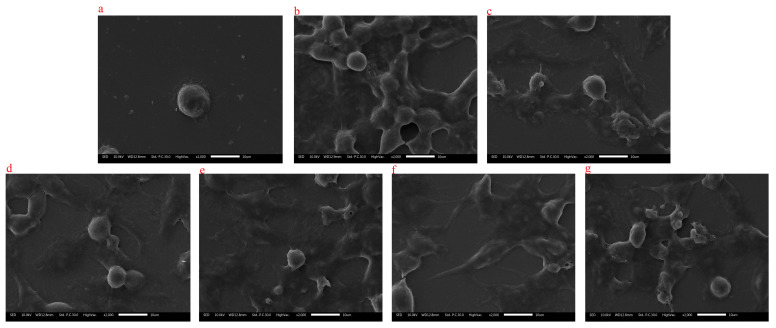
Scanning electron microscope results of the effect of MAC RAW264.7 cells (1000×, 2000×). Note: (**a**) was control group; (**b**) was lipopolysaccharide group; (**c**–**g**) were different concentrations of MAC (2, 4, 6, 8, and 10 µg/mL) group.

Scanning electron microscopy results are shown in Figure 8. The cells in the control group are round with clear borders and fewer pseudopods. Compared with the control group, the size of cell treated with LPS was larger, the shape was irregular and the antennae dendrite was increased. After the treatment of MAC, the cell showed similar morphology with LPS treatment. The results showed that MAC can activate macrophages to improve immunomodulatory effects.

*Misgurnus anguillicudatus* polysaccharide (MAP) also exerted its immunomodulating activity and promoted the greatest proliferation of spleen lymphocyte and macrophages in [27]. In general, the structure of polysaccharides may contribute to their immunomodulatory and other bioactivity [28]. Previous studies have also shown that the branched spherical structure of polysaccharides was beneficial to their bioactivity [29]. Therefore, the structures of MAO and MAP play an important role in their immune activity.

### 2.10. Effects of MAC on TNF-α and IL-6 Secretion in RAW264.7 Cells

Cytokines are intercellular signaling proteins released by immune and non-immune cells, and play a key role in controlling the balance of the whole organism [30]. In addition, in the treatment of cancer and autoimmune diseases, polysaccharides can up-regulate pro-inflammatory cytokines to avoid damage to patients caused by immunosuppression [31].

**Figure 9:**
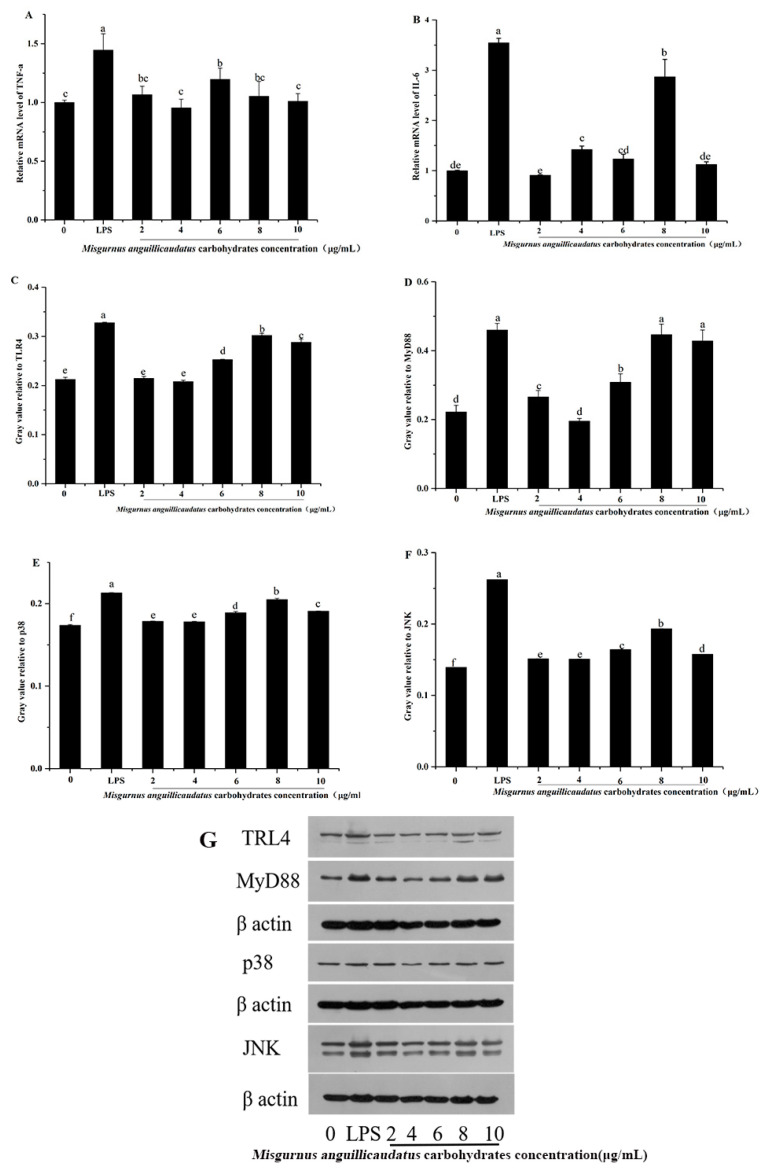
Effect of MAC on mRNA expression levels of TNF-α (**A**) and IL-6 (**B**) in RAW264.7 and the expression of TLR4, MyD88, and p38 protein (**G**) by WB. Density analysis (**C**–**F**) was represented by TLR4, MyD88, and p38 protein expression ratio to β-actin using ImageJ software (V1.8.0.112). Different letters indicate significant differences, *p* < 0.05.

It had been reported that different conformations of polysaccharides in solution, including single- and triple-helix, and random coil, can influence the direct contact between the polysaccharides and the cells or other components of the immune system. For instance, the authors reported that single-helix conformation had higher immunomodulatory activity to *Misgurnus anguillicudatus* polysaccharide via improving the viability of peritoneal macrophages, stimulating TNF-α and IL-6 production and inducing the inducible nitric oxide synthase (iNOS) [27]. To evaluate the effect of MAC on cytokines secreted by RAW264.7 macrophages, tumor necrosis factor-α (TNF-α) and interleukin-6 (IL-6) levels were detected by ELISA. In Figure 9A,B, MAC obviously induced the secretion of TNF-α and IL-6 in a dose-dependent manner (*p* < 0.05). Compared with the control group, the mRNA expression levels of IL-6 and TNF-α of RAW264.7 cells in the LPS group were significantly increased by 254.67% and 44.67%, respectively (*p* < 0.05). At 8 μg/mL, the expression of IL-6 mRNA was significantly increased by 187.00% (*p* < 0.05) and TNF-α mRNA was significantly increased by 19.67% (*p* < 0.05) compared to the control group. This result is consistent with the report of Chenxiao Zhang et al., which indicated that *Misgurnus anguillicudatus* polysaccharide stimulated TNF-α and IL-6 production [30]. It has been found that polysaccharides which mainly composed of arabinose, galactose, and rhamnose, are biological activity [32]. Li, J. et al. reported that the key roles in activation of macrophages are that of mannose, arabinose, xylose, and galactose, but not glucose [33]. This implies that arabinose present in MAO might contribute to its immunomodulatory activity.

### 2.11. Western Blot Analysis

Macrophage activation is mediated primarily by stimulating pattern recognition receptors (PRRS) that bind to polysaccharides, such as Toll-like receptors (TLR2 and TLR4) [34]. Some research reported that TLR4 mediates the involvement of polysaccharides in the immune system [35]. Therefore, in the present study, the roles of TLR4 on immunomodulatory activity of MAP were investigated. In Figure 9C, at 8 μg/mL, the expression levels of TLR4 protein was significantly increased by 42.18% (*p* < 0.05) compared to the control group. Therefore, we speculated that the MAC could through the activation of TLR4 to induce the immunomodulatory activity.

Toll-like receptors ligation leads to the activation of IL-1R-associated kinase (IRAK) via an adaptor myeloid differentiation protein 88 (MyD88), with subsequent activation of TNF receptor-associated factor 6 (TRAF-6), Jun N-terminal kinase (JNK) and NF-κB [36]. In this study, compared with the control group, the expression levels of protein MyD88, p38, and JNK were significantly increased by 107.42%, 22.59%, 88.13% (*p* < 0.05) in LPS-treated groups. Moreover, at 6, 8, and 10 μg/mL, the protein expression of MyD88 was significantly increased by 39.08%, 101.39%, and 93.17%, p38 was significantly increased by 8.73%, 17.89%, and 9.78%, and JNK was significantly increased by 17.88%, 38.74%, 13.24%. Taken together, we can conclude that MAC possessed the typical immunomodulatory activity.

**Figure 10:**
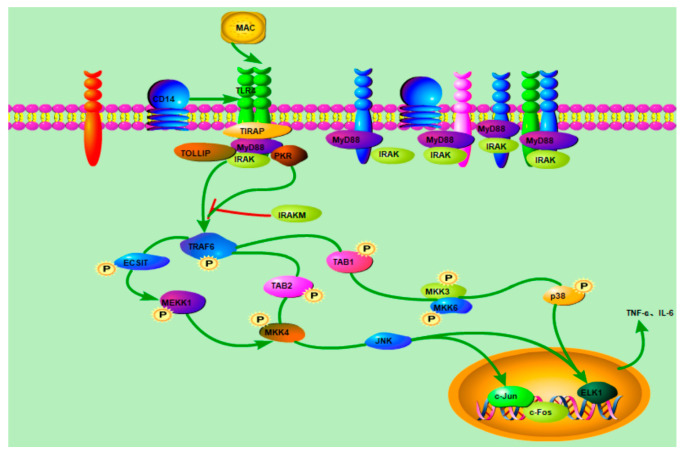
The speculated signaling pathway of immunomodulatory activity of *Misgurnus anguillicaudatus* carbohydrates.

These results showed that MAC, as an animal-derived polysaccharide, has a significant immunomodulatory activity. The immunomodulatory activity of polysaccharides mainly depends on its structural characteristics, such as monosaccharide composition, molecular weight, glycosidic bond and triple helix conformation [37]. Ai, J. et al. reported that polysaccharides with a low molecular weight and linear α-(1→4)-Glcp backbone have a high immunomodulatory activity [38], and usually promote immunomodulatory activity by the production of cytokines by macrophages [39]. Consequently, the immunomodulatory activity of MAC may be related to its lower molecular weight promoting the production of cytokines by macrophages. According to this study, with the speculated pathways characterized in Figure 10, it can be included that MAC can activate RAW264.7 cells by up-regulating the expression of cell surface receptor protein TLR4, causing the expression of MyD88, p38 and JNK in a dose-dependent manner.

## 3. Materials and Methods

### 3.1. Materials

Loach were purchased from Tianjin WangDingDi Agricultural Trade Market, Tianjin, China; C dimethyl sulfoxide and Sephadex G-100 were purchased from Beijing Soleibao Technology Co., Ltd. (Beijing, China); high-sugar DMEM medium supplemented with 100 IU/mL penicillin and 100 μg/mL streptomycin, fetal bovine serum, neutral red, RAW 264.7 cell, and 3-(4,5-dimethylthiazol-2-yl)-2,5-diphenyl tetrazolium bromide (MTT) were purchased from Beijing Dingguo Changsheng Biotechnology Co., Ltd. (Tianjin, China). Lipopolysaccharide was purchased from Shanghai Yuanye Biotechnology Co., Ltd. (Shanghai, China). Assay kits for interleukin-6 (IL-6), tumor necrosis factor-α (TNF-α) were purchased from Nanjing Jiancheng Bioengineering Institute (Nanjing, China). Rabbit anti-murine TLR4, MyD88, JNK, p38, and β-actin antibody were purchased from Abcam (Beijing, China). All other reagents were of analytical grade.

### 3.2. Extraction and Purification of Misgurnus anguillicaudatus Carbohydrates in Loach Mucus

Loach was minced and dried in oven at 50 °C. The powder was crushed and screened through 40 mesh, defatted with soxhlet-extracter and stored in dryer for further analysis. The degreased powder was extracted with water at a ratio of 1:5 (*w*/*v*) for 83 min at 59 °C for 3 times. The extract was centrifuged at 4000× *g* for 15 min, and the supernatant was collected and concentrated to 60 °C. The Sevag method was used to remove protein and was precipitated with four volumes of 100% ethanol at 4 °C for overnight. The precipitate was collected and extensively dialyzed (MW cut-off, 3500) against tap water for 24 h and distilled water for 24 h, then lyophilized to obtain crude *Misgurnus anguillicaudatus* carbohydrate (MAC). The total neutral sugar content was detected using the phenolsulfuric acid method with glucose as the standard. The soluble protein was determined according to the Coomassie brilliant blue G-250 method using bovine serum albumin (BSA) as the standard. Using galacturonic acid as the standard, the content of total glucuronic acid was determined by m-hydroxydiphenyl colorimetry method [13].

The crude MAC was dissolved and applied to a DEAE-52 cellulose column (1 cm × 20 cm) with distilled water and NaCl solution in different concentrations (0.1–0.4 mol/L) as eluent at a flow rate of 1 mL/min. According to the elution curve, two major fractions were eluted, respectively, with distilled water and 0.1 mol/L NaCl, and collected and dialyzed for further analysis.

### 3.3. Molecular Weight Determination

High-performance liquid chromatography (Shimadzu Corporation,LC-20, Kyoto, Japan) with a Shodex 802 liquid chromatographic column (Φ300 × 7.5 mm, Thermo Fisher, Kyoto, Japan) and a refractive index detector, eluted with ultrapure water at a flow rate of 1.0 mL/min was used to determine the molecular weight. Dextrans with different weight-average molecular weight (1000 Da, 4000 Da, 5000 Da, 7000 Da, and 16k Da) were used as standards for calculated. According to the molecular weight, the two parts were named as *Misgurnus anguillicaudatus* oligosaccharide (MAO) and *Misgurnus anguillicaudatus* polysaccharides (MAP), respectively.

### 3.4. Monosaccharide Composition Analysis

Monosaccharide composition was measured using gas chromatography (GC) as our previously described method [40].

### 3.5. FT-IR Analysis

One milligram of MAO or MAP was ground with 150 mg of potassium bromide powder and then pressed into pellets for FT-IR measurement (Thermo Nicolet, Waltham, MA, USA). The test frequency was 4000 cm^−1^ to 400 cm^−1^ and the scans number was 16 times.

### 3.6. Nuclear Magnetic Resonance (NMR) Detection

The samples were accurately weighed and dissolved in 0.5mL D_2_O and placed at room temperature for 5 h. The samples were loaded into a nuclear magnetic tube with an inner diameter of 5 mm using a pipette. The carbon spectra (^13^C NMR) were measured by an on-machine nuclear magnetic resonance instrument at 25 °C (resolution 400 MHz). The carbon spectrum was scanned 16 times with a bandwidth of 56.8 microseconds.

### 3.7. Congo Red Experiment

Two milliliters of both the MAO and MAP solutions (1.0 mg/mL) were mixed with 2 mL of Congo red (80 μmol/mL). After that, the resulting mixtures were further mixed with different concentrations of NaOH solution (0 mol/L, 0.1 mol/L, 0.2 mol/L, 0.3 mol/L, 0.4 mol/L, and 0.5 mol/L). The mixture was analyzed with 80μmol/L Congo red solution as a blank control. The λmax values of the Congo red-carbohydrate solutions at different NaOH concentrations were used to evaluate the transition from a triple helical preparation to a single stranded conformation.

### 3.8. Scanning Electron Microscopy (SEM)

A scanning electron microscope (SEM) was used to analyze the morphology features. The SEM images (1000×, 3500×, and 5000× magnifications) were analyzed with a Hitachi SU-1510 system (Hitachi, Pleasanton, CA, USA).

### 3.9. Immunomodulatory Activity of Misgurnus anguillicaudatus Carbohydrates (MAC)

#### 3.9.1. Cell Culture

RAW264.7 cells were cultured in DMEM high-glucose medium containing 10% fetal bovine serum (FBS), 100 U/mL penicillin and 100 μg/mL streptomycin in a humid 5% CO_2_ atmosphere incubator at 37 °C.

#### 3.9.2. Macrophage Proliferation Assay

The effect of MAC on the proliferation of RAW264.7 cells was determined by MTT assay [41]. One hundred microliters of RAW264.7 cells (5 × 10^4^ cells/mL) were plated in a 96-well plate concentration and then cultured with different concentrations of MAC (0, 2, 4, 6, 8, and 10 µg/mL) for different periods of time (24 h, 36 h and 48 h, respectively) at 37 °C in a humidified 5% CO_2_ atmosphere incubator. After incubation, 5 mg/mL MTT solution was added to each well. The plates were further incubated for 4 h. The produced formazan crystals were dissolved with 150 µL DMSO and shook at room temperature for 10 min. The absorbance at A490 nm of each well was determined by a microplate reader.

#### 3.9.3. Phagocytic Assay

The phagocytic ability of macrophages was measured by neutral red uptake [42]. One hundred microliters of cells (5 × 10^4^ cells/mL) was plated in a 96-well plate concentration. The cells were cultured with different concentrations of MAC (0, 2, 4, 6, 8, and 10 µg/mL) or LPS (10 µg/mL) for 24 h, 36 h and 48 h, respectively, at 37 °C in a humidified 5% CO_2_ atmosphere incubator. After that, 100 µL neutral red solution (dissolved in 10 mmol/L PBS with a concentration of 0.1% *w*/*v*) were added and incubated for 4 h in the dark. The absorbance value of each well at 540 nm was determined with a microplate reader.

#### 3.9.4. Scanning Electron Microscope Observation Assay

Two milliliter of RAW264.7 cells (5 × 10^4^ cells/mL) were plated in a 6-well plate concentration and then cultured with different concentrations of MAC (0, 2, 4, 6, 8, and 10 µg/mL) or LPS (10 µg/mL) for 36 h at 37 °C in a humidified 5% CO_2_ atmosphere incubator. After cultured, the supernatant was discard and washed 3 times with phosphate buffer, then fixed with 2.5% glutaraldehyde solution for 2 h and dewatered with different concentrations of ethanol (10%, 30%, 50%, 70%, 90%, 90%, and 100%). The external morphological changes were observed by a scanning electron microscope.

#### 3.9.5. The RT-PCR Method to Measure the Expression Level of Cellular Immune Factor mRNA

The RAW264.7 cells (5 × 10^4^ cells/mL) were cultured with different concentrations of MAC (0, 2, 4, 6, 8, and 10 µg/mL) or LPS (10 µg/mL) for 36 h at 37 °C in a humidified 5% CO_2_ atmosphere incubator. After cultivation, the supernatant was collected and ELISA kits were utilized to detect the immune factors. The sequence of qRT-PCR primers is shown in Table 3. The amplification procedure was: pre-denaturation at 94 °C for 2 min, denaturation at 94 °C for 30 s, and annealing/extension at 60 °C for 1 min, 35 cycles.

#### 3.9.6. Western Blot Assay

The RAW264 cell treatments were the same as in Section 3.9.5. During cultivation, the supernatant was collected and extracted with lysis buffer to obtain total cell protein. Western blot was used to detect the protein expression level of RAW264.7 cells. The β-actin was the internal control.

### 3.10. Statistical Analysis

SPSS software (version 22.0, SPSS Inc., Chicago, IL, USA) was used to evaluate the data, and ANOVA was used to analyze the statistical significance of differences between groups. *p* > 0.05 and *p* > 0.01 was considered statistically significant and very significant, respectively. The experimental data were expressed as the mean ± standard deviation.

## 4. Conclusions

In this study, two new heterosaccharides (MAO and MAP) with a molecular weight of 2854 Da and 3873 Da, respectively, were separated from *Misgurnus anguillicaudatus*. MAO principally consisted of arabinose (77.11%), rhamnose (21.97%) and fucose (0.92%); MAP mainly was composed of fucose (87.55), rhamnose (8.86%) and galactose (3.59%). The results of in vitro cell experiments indicated that MAC has significant immunomodulatory activity, possibly by promoting the phagocytosis activity and enhancing the production of IL-6 and TNF-α. TLR4, MyD88, p38 and JNK were confirmed to be the major protein of MAC on RAW 264.7. It was therefore suggested that MAC could improve immunity through TLR4-MAPK-p38 signaling pathways. Consequently, these results show that the study of the structure and immune activity mechanism of MAC is important to the comprehensive development and utilization of loaches.

## Figures and Tables

**Table 1:** Molecular weight of MAO and MAP analyzed by HPLC.

Sample	Retention Time (min)	Mw (Da)	Mn (Da)	Mw/Mn
MAO	9.034	2854	2114	1.35
MAP	8.683	3873	2748	1.41

**Table 2:** The results of sample composition (MAC, MAO, and MAP).

Sample	Total Sugars Content (%)	Protein Content (%)	Glucuronic Acid Content (%)
MAC	76.31% ± 0.11%	23.53% ± 0.12%	0.97% ± 0.02%
MAO	94.432% ± 0.45%	-	-
MAP	98.80% ± 0.27%	-	1.08% ± 0.26%

Note: “-” mean not detected.

**Table 3:** qRT-PCR primer sequence.

	Forward	Reverse	Base Pair Length
TNF-α	CCTGTAGCCCACGTCGTAG	GGGAGTAGACAAGGTACAACCC	148 bp
IL-6	CTGCAAGAGACTTCCATCCAG	AGTGGTATAGACAGGTCTGTTGG	131 bp
β-actin	GCCTTCCGTGTTCCTACC	GGAGACAACCTGGTCCTCA	156 bp

## Data Availability

No new data were created.

## Sample Availability

Samples of the compounds MAC, MAO and MAP are available from the authors.
